# Starch Characteristics and Amylopectin Unit and Internal Chain Profiles of Indonesian Rice (*Oryza sativa*)

**DOI:** 10.3390/foods13152422

**Published:** 2024-07-31

**Authors:** Juan Giustra Mogoginta, Takehiro Murai, George A. Annor

**Affiliations:** Department of Food Science and Nutrition, University of Minnesota, 1334 Eckles Avenue, Saint Paul, MN 55108, USA; mogog001@umn.edu (J.G.M.); murai021@umn.edu (T.M.)

**Keywords:** rice, amylopectin, internal chain profile, unit chain profile

## Abstract

Indonesia is arguably a major player in worldwide rice production. Though white rice is the most predominantly cultivated, red, brown, and red rice are also very common. These types of rice are known to have different cooking properties that may be related to differences in their starch properties. Investigating the starch properties, especially the fine structure of their amylopectin, can help understand these differences. This study aims to investigate the starch characteristics of some Indonesian rice varieties by evaluating the starch granule morphology and size, molecular characteristics, amylopectin unit and internal chain profiles, and thermal properties. Starches were extracted from white rice (long grain (IR-64) and short grain (IR-42)), brown rice, red rice, and black rice cultivated in Java Island, Indonesia. IR-42 had the highest amylose content of 39.34% whilst the black rice had the least of 1.73%. The enthalpy of gelatinization and onset temperature of the gelatinization of starch granules were between 3.2 and 16.2 J/g and 60.1 to 73.8 °C, respectively. There were significant differences between the relative molar amounts of the internal chains of the samples. The two white rice and black rice had a significantly higher amount of A-chains, but a lower amount of B-chains and fingerprint B-chains (B_fp_) than the brown and red rice. The average chain length (CL), short chain length (SCL), and external chain length (ECL) were significantly longer for the red rice and the black rice in comparison to both the white rice amylopectins. The long chain length (LCL) and internal chain length (ICL) of the sample amylopectins were similar. Rice starches were significantly different in the internal structure but not as much in their amylopectin unit chain profile. These results suggest the differences in their amylopectin clusters and building blocks.

## 1. Introduction

Rice is a staple grain around the world, and its use for food is projected to reach 426.5 million tonnes in the 2022/2023 season [1]. India and China account for about 50% of paddy rice production, followed by Indonesia [2]. According to the International Rice Research Institute, the production of milled rice in Indonesia was 70 million metric tonnes. Rice belongs to the Gramineae or grass family along with barley, wheat, corn, rye, and oats. There are mainly two species of rice produced, namely *Oryza sativa* (Asian rice) and *Oryza glaberrima* (African rice), where Indonesian rice is mainly *Oryza sativa.*

Rice is classified as white rice or colored rice (such as brown, red, or black rice). While white rice is milled and polished to remove the outer layer (husked and bran), colored rice still has the bran intact [3]. White rice can also be called milled or polished rice, while colored rice can be called husked rice [4].

The composition of rice can vary depending on its type—white or husked. White rice and husked rice both contain about 5–15% protein [5,6,7], 79–83% carbohydrate, and 0.48–1.1% crude fiber [8,9]. White rice (0.4–0.6%) has a lower mineral content than husked rice (1.7–1.9%) due to the bran being removed during the milling. Husked rice, especially black and red rice, has higher polyphenol, tannins, and antioxidant activity compared to white rice [10,11]. In addition, the total content of soluble and insoluble phenolic acid in brown rice is higher than white rice [12]. The total phenols present in colored rice have several health benefits, such as anticarcinogenic, antioxidant, cardiovascular, and cholesterol-lowering properties. However, tannins that exist in higher amounts in husked rice can decrease the digestibility of proteins, carbohydrates, and minerals [13].

Starch is made of amylopectin and amylose. Amylopectin is the major component of starch that dictates starch behavior such as starch gelatinization [14,15]. However, in rice, amylose content is also important as it affects the cooking quality of rice. Rice can be divided into subcategories according to its amylose content: high 25–33%, intermediate 20–25%, very low 5–10%, and waxy 0–2% [16]. On average, rice amylose has a degree of polymerization (DP) value of 980–1110, average chain lengths of 250–370, and a slightly branched molecule of 2–5 chains [17]. The chains of amylopectin can be divided into two parts: internal chain and external chain. The internal chain forms the backbone of the amylopectin consisting of B-chains and C-chains [18,19]. The external chains are also called A-chains and do not carry other chains. B-chains are involved in both the external and internal chains as they serve to structurally connect the A- and C-chains [20]. A-chains are shorter chains with a DP of 6–12, while C-chains are longer with a DP greater than 36. B-chains are further categorized into B1 (DP 20–24), B2 (DP 42–48), and B3 (DP 69–75) [21]. A-chains are found in crystalline lamella in the starch granules forming double helices. B-chains are found mainly in amorphous lamella, with branches that overlap in the crystalline lamella [22].

Amylopectin unit and internal chain have been analyzed for their size distribution with high-performance anion-exchange chromatography (HPAEC) in the past. The α-(1,6) linkages of amylopectin are first hydrolyzed with isoamylase and pullulanase and sequentially treated with phosphorylase *a* and β-amylase to remove the external chains. The successive addition of both phosphorylase *a* and β-amylase produces φ,β-limit dextrins in which A-chains appear as maltosyl units and external chains of the B-chains appear as three or more glucosyl units [23]. Bertoft and others [24] divide B-chains into short chains (BS) and long chains (BL). BS-chains are further divided into two groups, “fingerprint” B_fp_-chains (DP 3–7), and BS_major_-chains (DP 8–25). B_fp_-chains are the characteristic profile for different plant samples [18]. The unit and internal chain structure of amylopectins from different botanical sources has been previously reported [24,25,26,27,28,29,30,31,32].

In this study, starch extracted from five Indonesian rice varieties [two white rice (IR-64 and IR-42) and three husked rice (brown rice, red rice, and black rice)] were fractionated into amylopectin and amylose, and their unit and internal chain profiles were studied. The molecular and physical characteristics of the native starches were also studied. The objective of this study was to determine the differences in the amylopectin unit and internal chains of these rice varieties in relation to starch’s physical and molecular properties. 

## 2. Materials and Methods

### 2.1. Materials

The five rice samples used for the study were long-grain white rice (IR-64), short-grain white rice (IR-42), brown rice, red rice, and black rice to represent the rice varieties that are commercially obtainable in Indonesia. The rice samples were obtained from PT. Tiga Pilar Sejahtera (Sragen, Central Java, Indonesia). The rice samples were harvested in the third quarter of 2016 and stored at 4 °C. The brown, red, and black rice were used whole for further analysis.

### 2.2. Starch Extraction

The rice kernels were frozen with liquid nitrogen and then milled for 1 min into flour with a Hamilton Beach Fresh-Grind Coffee Grinder (Hamilton Beach Brands, Glen Allen, VA, USA). The starch was extracted from the flour samples based on the method by Waduge and others [33] with modifications. The extraction buffer solution (12.5 mM, pH 10, containing 0.5% SDS and 0.5% Na_2_S_2_O_5_ (*w*/*v*)) was added to the mixture with a ratio of 20 volumes per 1 g of flour. The mixture was stirred for 10 min and recovered by centrifugation at 3600× *g* for 10 min (4 °C). The extraction process was repeated. The resulting residue was washed three times with distilled water and recovered with centrifugation at 3600× *g* for 10 min (4 °C). The residue was then suspended in distilled water and the starch slurry was passed through four layers of cheesecloth and then through a 70 μm nylon mesh. The slurry was centrifuged at 3600× *g* for 10 min (at 4 °C). The brown layer on top of the starch layer was removed with a spatula. The above processes were repeated until all the brown particles were removed from the starch fraction. The pellets were collected and washed with acetone, shaken hard, and recovered by centrifuge at 3600× *g* for 10 min (4 °C). The pellets were air-dried under a fume hood overnight.

### 2.3. Amylopectin Fractionation

Approximately 2 g of rice starch was dissolved in 40 mL of 90% DMSO and boiled for 10 min. The samples were then stirred overnight at room temperature (25 °C). The samples were then boiled for 10 min before 4 volumes of ethanol were added into the mixtures. The samples were then cooled to room temperature and centrifuged at 8000× *g* for 10 min (4 °C). The supernatant was discarded, and the pellet was dissolved with hot double-distilled water (80 °C). The pellet was then washed again to collect the non-granular portion of the starch. The non-granular starch was dissolved with 56 mL 90% DMSO and boiled for 3 h and stirred until totally dissolved. The sample was combined with a mixture of 1-butanol (23.5 mL), isoamylalcohol (23.5 mL), and water (324 mL). The mixture was then cooled at room temperature (25 °C) for 20 h. The sample was centrifuged at 22,000× *g* (4 °C) for 30 min. The supernatant was then reduced to 50 mL and a mixture of 1-butanol, isoamylalcohol, and water was added. The supernatant was reduced to 50 mL, and three volumes of methanol was added to the amylopectin solution. The sample was left overnight. The mixture was then centrifuged at 22,000× *g* at 4 °C for 30 min, and the precipitate was dissolved in hot double-distilled water (80 °C). The 4× volume of ethanol was then added and the precipitate from the centrifugation was dissolved in hot water (80 °C). The amylopectin solution was then freeze-dried at −48 °C under vacuum overnight.

### 2.4. Thermal Properties

The gelatinization properties of the samples (3 mg) were measured with a differential scanning calorimeter Q1000 equipped with thermal analysis data and a recording software facility (TA Instruments, Universal Analysis 2000, New Castle, DE, USA). The samples were dispersed in water (1:3) and equilibrated for at least 3 h at room temperature (25 °C). The thermograms were recorded for the onset (T_o_), peak (T_p_), and conclusion (T_c_) transition temperatures with an empty aluminum pan as a reference. The temperature range was programmed to increase from 20 to 120 °C at a heating rate of 10 °C/min. The enthalpy of gelatinization (ΔH) expressed as J/g of dry starch was estimated by integrating the area between the thermogram and the base line under the peak. 

### 2.5. Size Distribution of Debranched Starches

**Figure 1:**
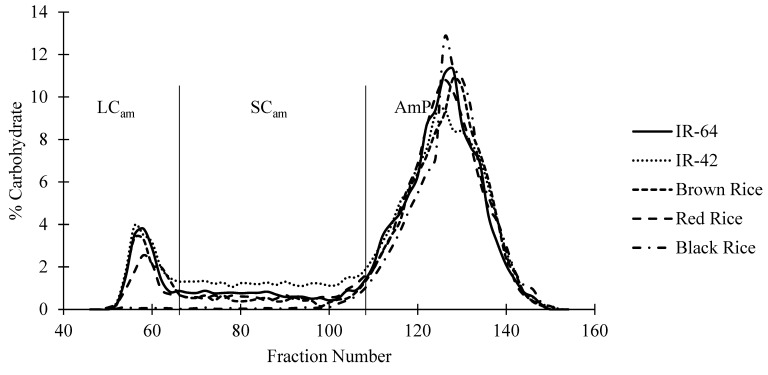
Sepharose CL-6B gel-permeation chromatogram of debranched rice starches. LC_am_ = long-chain amylose, SC_am_ = short-chain amylose, and AmP = amylopectin chains.

The starch samples (6 mg) were dissolved in 90% dimethyl sulfoxide (DMSO; 150 μL) and heated in a water bath (80 °C) for 5 min and stirred slowly overnight at room temperature (25 °C). Subsequently, water (900 μL, at 80 °C) was added to the sample and left to cool to room temperature (25 °C). Approximately 100 μL of 0.01 M sodium acetate buffer (pH 5.5), 1 μL of isoamylase enzyme (Hayashibara, Okayama, Okayama, Japan), and 1 μL of pullulanase M1 were added directly. The tubes were then stirred slowly overnight at room temperature (25 °C). The samples were placed in a water bath (80 °C) for 5 min to inactivate the enzyme. The sample (1 mL) was then injected into a Sepharose CL-6B gel column (1 × 90 cm, GE Healthcare, Uppsala, Sweden), and eluted with 0.5 M NaOH at 1 mL/min. Void solutions of NaOH (40 mL) were eluted before each sample. The fractions (1 mL) were analyzed for carbohydrates with a phenol–sulfuric acid reagent [34]. The analysis was conducted by taking the absorbance of the fraction and phenol–sulfuric acid reagent under a spectrophotometer (Shimadzu UVmini-1240, Kyoto, Japan) at a wavelength of 490 nm. The relative amounts of amylose and amylopectin are shown by the two peaks in Figure 1. The first peak is the amylose and the second is the amylopectin. The long-chain amylose is eluted before the short-chain amylose and amylopectin is the last to be eluted [35].

### 2.6. Size Distribution of Starch Components and Their β-Limit Dextrins

The starch samples (6 mg) were dissolved in 90% DMSO (150 μL) and heated in water (80 °C) for 5 min and stirred slowly at room temperature (25 °C) overnight. The samples were then diluted with warm water (1.85 mL, at 80 °C). The size distribution of the starch components was chromatographed on a Sepharose CL-2B column (1.6 × 32 cm) (Pharmacia, Uppsala, Sweden). Seven (700) microliters of starch solution was eluted through the column with 0.01 M NaOH at a rate of 0.5 mL/min. Approximately 1 mL of fractions were collected and separated into two tubes (an odd tube and an even tube), and the odd tube was tested for the carbohydrate content, determined by the phenol–sulfuric acid reagent [34]. The even tube was tested for the wavelength maxima (λ_max_) of the glucan–iodine complex. A total of 1 mL of 0.01 M HCl was added to neutralize the fractions, and then 0.1 mL of 0.01 M I_2_/0.1 M KI solution was added. The absorbances of the phenol–sulfuric acid reagent and glucan–iodine complex were determined with a WPA Spectrawave S800 diode array spectrophotometer (Biochrom Ltd., Cambridge, UK).

The β-limit dextrins (β-LDs) of the rice starches were produced according to the method reported by Bertoft [36]. The starch samples (6 mg) were dissolved in 90% dimethyl sulfoxide (DMSO; 150 μL) and heated in a water bath (80 °C) for 5 min and then stirred slowly overnight at room temperature (25 °C). The next day, warm water (900 μL, at 80 °C) was added to the sample and cooled to room temperature. Approximately 100 μL of 0.01 M sodium acetate buffer (pH 6) and 2 μL of β-amylase (4 U/mg) (Megazyme, Bray, Ireland) was added directly. The tubes were then stirred slowly overnight at room temperature (25 °C). The samples were placed in a warm water bath (80 °C) for 5 min to inactivate the enzyme. In total, 700 μL of the mixture was injected into the Sepharose CL-2B column and analyzed on HPAEC. 

### 2.7. Production of φ,β-Limit Dextrins

φ,β-limit dextrins were produced based on the method by Bertoft [36] with modifications by Kalinga and others [37]. 

### 2.8. Analysis of Unit Chain Distributions of Amylopectins and Their φ,β-Limit Dextrins

The analysis of the unit chain distributions of amylopectins and their φ,β-limit dextrins was based on the method from Annor and others [26] with modification. Approximately 2 mg of amylopectin or their φ,β-limit dextrins was dissolved in 50 μL of 90% DMSO with constant stirring overnight until dissolved. The solution was then diluted with 400 μL of warm water (80 °C). The sample was cooled down to room temperature and 1 μL pullulanase M1 and 1 μL isoamylase (Megazyme International, Bray, County Wicklow, Ireland) were added subsequently. The sample was stirred overnight in order to debranch the amylopectin and their φ,β-limit dextrins. The enzymes were then inactivated by boiling for 5 min and the sample was then diluted into a concentration of 1 mg/mL. The sample was filtered using a 0.45 μm nylon filter and 25 μL was injected into the Dionex ICS 3000 high-performance anion-exchange chromatography (HPAEC) system (Dionex Corporation, Sunnyvale, CA, USA) equipped with a pulsed amperometric detector (PAD), CarboPac PA-100 ion-exchange column (4 × 250 mm), and the corresponding guard column (4 × 50 mm). The DIONEX was then run at a flow rate of 1 mL/min and eluent A was 150 mM sodium hydroxide and eluent B was 150 mM sodium hydroxide with 500 mM sodium acetate. The samples were run for 130 min, divided into five stages with different ratios of eluents A and B. During the first 9 min, the concentration of eluent B increased from 15% to 36%. From 9 to 18 min, the concentration of eluent B was increased to 45%. From 18 to 110 min, the concentration of eluent B was further raised to 100%. This was followed by a quick decrease to 15% from 110 to 112 min. For the final 18 min, the eluent B level was maintained at 15%. The chromatograms were then converted into carbohydrate division using the method by Koch et al. (1998) [38].

### 2.9. Starch Granule Microscopy

All the samples were observed with the Hitachi S-570 scanning electron microscope (SEM, Hitachi Scientific Instruments, Rexdale, ON, Canada). The samples were mixed with 15 nm of gold dust on a stub and the working distance used was 15 mm with a voltage of 10 kV.

### 2.10. Statistical Analysis

Statistical analysis was conducted using R 3.5.1 with a one-way analysis of variance (ANOVA) at a confidence level of 95%. A post hoc test was conducted using Tukey’s HSD method.

## 3. Results and Discussion

### 3.1. Amylose Content and Chain Profile

The amylose contents of the rice starches were determined by analyzing the chain profile of the debranched rice starches with Sepharose CL-6B gel-permeation chromatography (Figure 1). The values were 20.68, 25.51, 20.23, 18.00, and 1.64% for IR-64, IR-42, brown rice, red rice, and black rice starches (Table 1). In comparison, the other varieties of rice in Indonesia such as Pandan Wangi and Rojolele have amylose contents of 25.9% and 24.6%, respectively [39]. The long-chain (LC_am_) and short-chain (SC_am_) amylose ratio can be found in Table 1. All the samples except for the black rice had a higher amount of LC_am_ compared to the SC_am_. A higher ratio of LC_am_ to SC_am_ reflects a higher amount of the long chains of amylose, and on the other hand, a lower ratio of LC_am_ to SC_am_ reflects a higher amount of the short chains of amylose. The ratio of LC_am_ to SC_am_ is different compared to the value reported by Gayin and others [15]. The LC_am_ to SC_am_ ratios of IR-64, IR-42, brown rice, red rice, and black rice were 1.37, 0.89, 2.44, 1.11, and 0.41, respectively.

### 3.2. Size Distribution and Iodine Binding Characteristic

**Figure 2:**
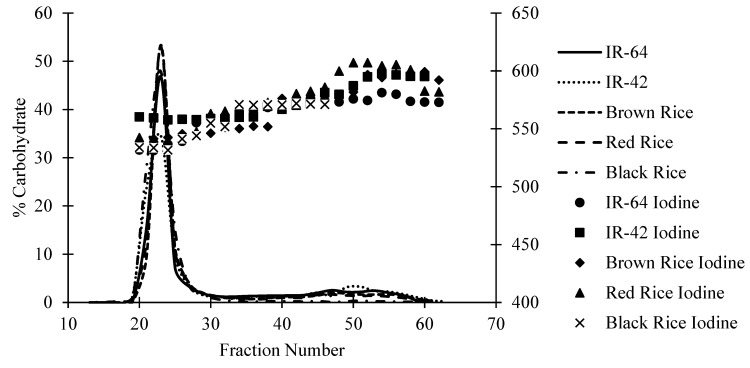
Sepharose CL-2B gel-permeation chromatograms of whole rice starches. The lines represent the carbohydrate content and the symbols represent the λ_max_ values.

The size distribution and iodine-binding characteristics of the starches shown in Figure 2 are represented as the percentage of carbohydrate and λ_max_ values, respectively. A higher λ_max_ value indicates longer chains that are able to bind with iodine more effectively. The gel-permeation chromatograms of the rice starches in Figure 2 are divided into two peaks. The first peak represents amylopectin, whilst the smaller, second peak represents amylose. The amylopectin portion recorded similar values of λ_max_. The λ_max_ values of amylopectin ranged from 532 to 565 among the rice samples, where the red rice had the highest λ_max_ value of 565 and the black rice with the lowest λ_max_ value of 532. Amylopectin binds with iodine through both the external and internal chains [40]. The amylopectin content of the black rice was significantly higher than the rest of the samples, and it had the shortest chain of amylopectin. The amylose also showed the same results, where the red rice had the longest chain of amylose with a λ_max_ of 607, while the black rice had a λ_max_ of 571.

### 3.3. β-Limit Dextrin

**Figure 3:**
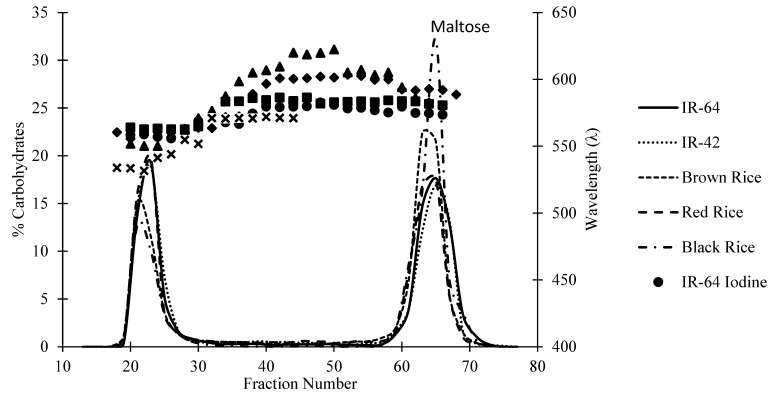
Sepharose CL-2B gel-permeation chromatograms of the rice starch β-limit dextrins. The lines represent the carbohydrate content, and the symbols represent the λ_max_ values.

The β-limit dextrins of the Indonesian rice starches fractionated on the Sepharose CL-2B along with their λ_max_ values are shown in Figure 3. The figure shows two different peaks, where the first peak is the β-limit dextrin and the second peak is the maltose peak. The maltose peak was generated by the addition of β-amylase to the starch samples which removed the linear polymers and external chains of the branched glucans. The λ_max_ values of the β-LDs from all the samples were observed to be similar or lower with their corresponding starches throughout the region. Similar to the λ_max_ values of the starch samples, the red rice had the highest values, while the black rice had the lowest value. The results contradict the finding of different starches like other rice starches [15]. This shows that there is an availability of internal glucan chains for iodine binding, and it is influenced by the branching pattern [15]. The difference in the λ_max_ values may be due to the differences in the average chain length and relative molar amount of the external and internal chain of its amylopectin.

### 3.4. Starch Granule Microscopy

**Figure 4:**
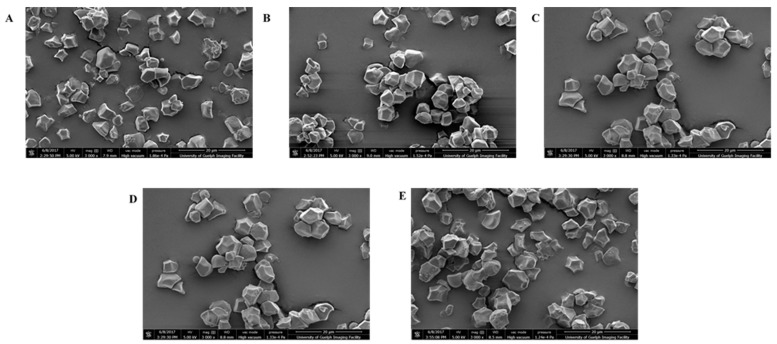
Scanning electron micrographs of rice starches. Magnification of 40 um ((**A**): IR-64 starch, (**B**): IR-42 starch, (**C**): brown rice starch, (**D**): red rice starch, and (**E**): black rice Starch).

The figure of the scanning electron micrographs shows similar sizes and shapes among the starch granules from the different samples (Figure 4). The Indonesian rice starch granules from this study had polygonal granular shapes, which agree with previous shapes from previous reports [41]. According to Wang and others [42], there are no significant differences in size, shape, and distribution between various Asian rice starches. The range of the Asian rice starch size for indica and japonica are 6.2–7.7 μm and 6.7–7.8 μm, respectively [42].

### 3.5. Thermal Properties

**Figure 5:**
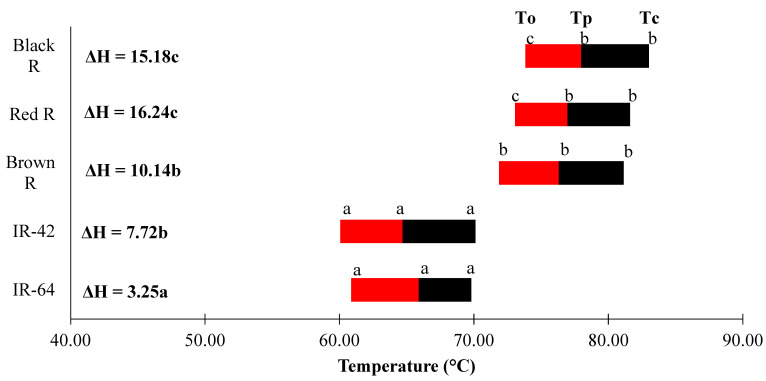
Gelatinization parameters of the rice starches. T_o_ = onset temperature; T_p_ = peak temperature; T_c_ = conclusion temperature; and ∆H = the enthalpy of gelatinization. The different letters are significantly different from each other.

The thermal properties of the rice starches differed between the husked rice and white rice (Figure 5). Starch gelatinization is the disturbance of the double helixes in the starch granules. These double helixes are found in amylopectin, but not in amylose [43]. The overall gelatinization temperatures of the Indonesian milled rice starches were significantly lower (*p* < 0.05) than the husked rice. The onset temperature (T_o_) of the black rice, red rice, brown rice, IR-42, and IR-64 was 73.83 °C, 73.06 °C, 71.88 °C, 60.88 °C, and 60.07 °C, respectively, in descending order. The peak temperature (T_p_) followed the same order as the onset temperature, ranging from 64.70 to 77.98 °C. However, the conclusion temperature (T_c_) showed a different pattern, where IR-42 (69.81 °C) had a lower conclusion temperature than IR-64 (70.13 °C) while all the husked rice starches had a T_c_ higher than 81.6 °C. Significant differences between enthalpies (∆H) were observed where the two white rice varieties, IR-42 and IR-64, had the lowest (∆H) of 7.72 J/g and 3.25 J/g, respectively. The brown rice had the lowest value among the husked rice with a ∆H of 10.14 J/g, black rice with a ∆H of 15.18 J/g, and red rice with a ∆H of 16.42 J/g. The results from this study differed from the previous studies of Indonesian rice starches and the same type of rice cultivated in different areas. According to Anugrahati and others [44], they found out that the starches they extracted had a lower onset temperature, similar peak temperature, and a higher conclusion temperature, and the enthalpies are also higher. The gelatinization temperature range was wider, and the enthalpies were high. On the other hand, IR-64 had a gelatinization temperature range from 71.8 to 82.4 °C with a ∆H of 5.1 J/g [45]. The difference from the results reported in the literature may be due to the environment in which the rice was grown. IR-64 which was cultivated in a different area, India, had a higher gelatinization temperature and slightly higher enthalpies. The husked rice had a higher temperature of gelatinization than the white rice. This proposes that it has a more perfect crystalline structure of amylopectin. The enthalpies of the gelatinization of the husked rice were higher than the white rice. This suggests a greater amount of longer double helices in the external chains of the amylopectins found in the husked rice [46]. The variations observed in the gelatinization temperatures of the starches from different rice might be due to the differences in the internal structure of amylopectin [47].

### 3.6. Unit Chains Profile

**Figure 6:**
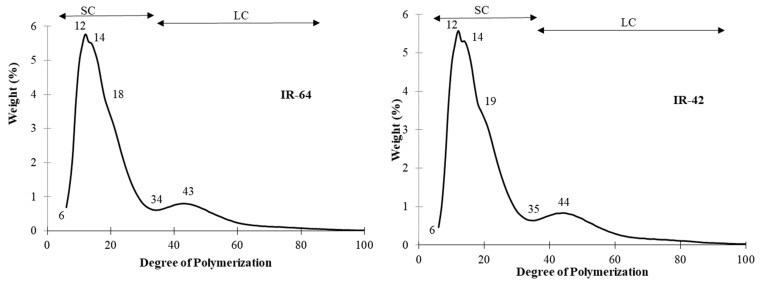

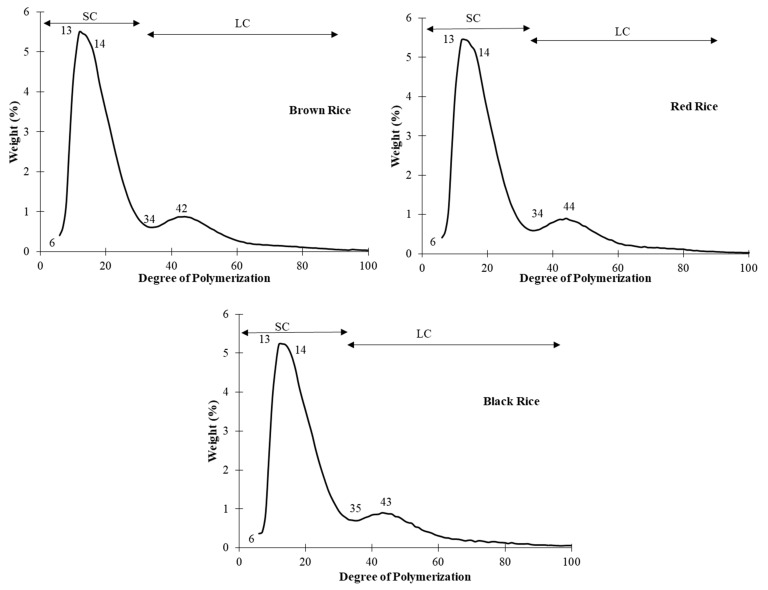
The unit chain profile of the debranched rice amylopectins by high-performance anion-exchange chromatography. The chain categories and DP values are indicated. SC = short chains; LC = long chains.

The unit chain profiles of the rice amylopectins investigated with HPAEC are shown in Figure 6. The rice amylopectins showed two different profiles. The peak patterns of all five samples were similar where there was an initial peak at DP 12–13 and a second peak at DP 14. The second peak at DP 14 was more prominent in the white rice than the brown, red, and black rice (husked rice). The white rice possessed a shoulder at DP 18–19, but not in the husked rice. It is similar to the results reported previously except for the husked rice [31,48]. The division between the short chain and long chain was different between the samples. Three of the samples, IR-64, brown rice, and red rice, were divided at DP 34, and the rest at DP 35. The peak for the long-chain amylopectin of all five samples falls between DP 42 and 44.

**Figure 7:**
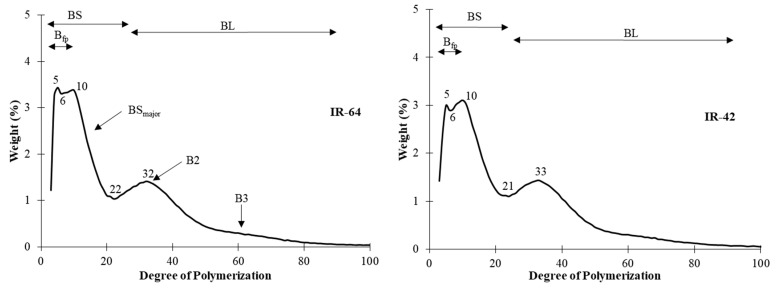

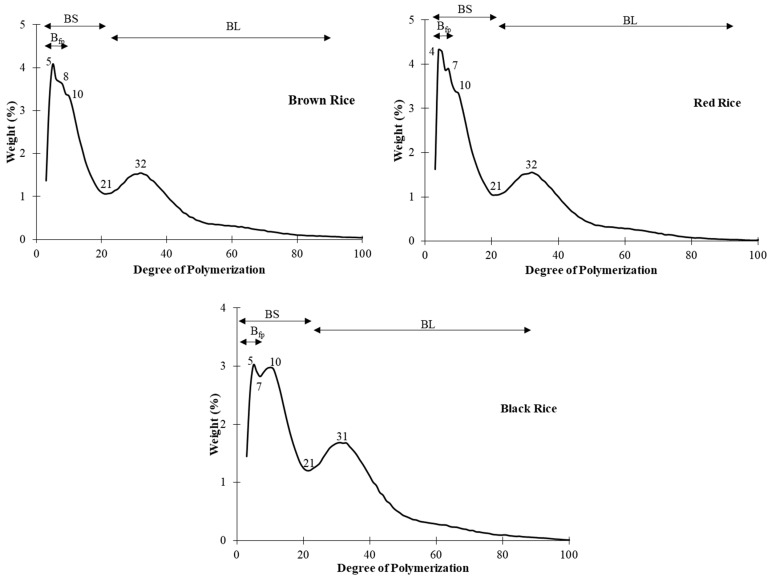
The unit chain profile of the debranched φ,β-limit dextrins of the rice amylopectins obtained by high-performance anion-exchange chromatography. BS = short B-chains are subdivided into “fingerprint” B-chains (B_fp_) and a major group (BS_major_). BL = long B-chains are subdivided into B2-chains and B3-chains.

Figure 7 shows the unit chain profile of the φ,β-limit dextrins of rice amylopectins obtained by HPAEC. The figure displays the distribution of the B-chains of φ,β-limit dextrins, showing the internal chains profiles of amylopectin. The division between the BS-chains and BL-chains of all the samples was the same at DP 21, except for IR-64 which was at DP 22. Bertoft and others [24] suggested that short B-chains have two distinct subgroups, the major group being DP 8–25, and the minor being DP 3–7. The B_fp_ of the samples fell between DP 4 and 5. The rest of the BS-chain is called BS_major_ chain. While BS_major_ chains are found at DP 10, the brown rice and red rice had an extra peak at DP 7 and 8, respectively. IR-64, brown rice, and red rice had the same peak of BL-chains at DP 32, whereas the black rice and IR-42 had the peak at-values at DP 31 and 33, respectively. The peak included the major sub-group B2-chains, and B3-chains with DP > 55 did not show any peak. This suggests that B3-chains were a minor category, which agrees with previous reports [24,27,31].

The average chain lengths and φ,β-limit values of rice amylopectin obtained from the chromatogram are shown in Table 2. The average chain length was 16.70–18.32, and the short-chain length was 14.55–15.54. These values are similar to other cereal grains reported previously [24]. The husked rice had a significantly longer chain length and short-chain length than the milled rice. The φ,β-limit value of rice amylopectins ranged from 57.24 to 60.35%. All three husked rice had a higher amount of φ,β-limit value than the two white rice. Annor and others report that the φ,β-limit value reflects the length of external chains [26]. The external chain length of white rice amylopectins was significantly lower than the external chain length of the husked rice amylopectins. This suggests that the husked rice may have a thicker lamella than the other samples because of the external chain present in the crystalline lamella of the starch granules.

Table 3 shows the relative molar amount of chain categories in the amylopectins of the rice starches. The A-chains of IR-64, IR-42, and black rice (53.98%, 53.84% and 53.12%) were significantly more than the brown rice and red rice (49.78% and 48.27%). However, the B-chains and B_fp_ of the brown rice and red rice were significantly higher than IR-64, IR-42, and black rice. According to Bertoft and Koch (2000) [25], B_fp_-chains participate in tightly branched building blocks in the clusters. All five samples had a similar BS_major_ of 19–20%. These BS_major_-chains are known to have interconnection between the backbones and external building blocks [49]. There was no difference in the molar amounts of B2- and B3-chains, where the B2-chains of the samples are between 4.90 and 4.99% and B3-chains between 0.64 and 0.82%. The results of BS_major_-, B2-, and B3-chains agree with the literature, where cereal grains have a high value of BS_major_, B2-chains, and a trace amount of B3-chains [24].

The molar ratio of the different chain categories of rice amylopectins and their φ,β-limit values are shown in Table 4. Bertoft and others [24] categorized different botanical amylopectins according to their internal chain profiles. These ratios together with the relative molar amounts of the different chain categories can help identify which type the samples fall into. Type 1, type 2, type 3, and type 4 are categorized by the ratio of BS:BL. Type 1 amylopectins have a high number of BS-chains and little BL-chains, resulting in a high ratio of BS:BL. The ratio decreases as follows: type 2 > type 3 > type 4. Other molar ratios characterize which type the amylopectins belong to as well [24]. Following the value of BS_major_-, B2-, and B3-chains from Table 3, the amylopectin followed the classification of type 2. Moreover, the molar ratios of S:L, A_crystal_:BS, A_crystal_:B, and B_fp_:BS_major_ fit the samples into type 2 [24,31].

## 4. Conclusions

This study showed the different compositions of starch and the amylopectin of the five Indonesian rice starches. The white rice exhibited differences in its starch gelatinization properties when compared to the husked rice. In this study, the white rice had a significantly lower overall temperature of gelatinization than the husked rice, and the enthalpies of the gelatinization of the milled rice were significantly lower as well. Each rice was different in its internal structure but not in its amylopectin unit chain profile. Although the chromatogram of the unit chain profile of the debranched φ,β-limit dextrins of the brown rice and red rice amylopectins had three peaks similar to type 1 amylopectins, the molar ratio suggests that the brown rice and red rice are more similar to type 2 amylopectin. Both the red rice and brown rice had more B-chains and B_fp_ compared to the rest of the samples, while the black rice displayed the longest B-chains. These differences might suggest the reason why husked rice has a higher temperature of gelatinization and higher enthalpies of gelatinization. 

## Figures and Tables

**Table 1:** Amylose content and composition of rice starches.

Sample	Amylose (%)	LC_am_ (%)	SC_am_ (%)	LC_am_:SC_am_
IR-64	20.68 b	11.96 bc	8.72 b	1.37 c
IR-42	25.51 c	12.55 bc	12.96 c	0.89 b
Brown	20.23 b	14.36 c	5.88 b	2.44 d
Red	18.00 b	9.58 b	8.66 b	1.11 bc
Black	1.64 a	0.46 a	1.17 a	0.41 a

Values of amylose—SC_am_ are expressed as mean percentage (*n* = 2). Different letters on each column are significantly different (*p* < 0.05) from each other. According to one-way ANOVA. LC_am_ = long-chain amylose; SC_am_ = short-chain amylose.

**Table 2:** Average chain lengths and φ,β-limit values of rice amylopectin obtained by high-performance anion-exchange chromatography.

Sample	CL	SCL	LCL	ECL	ICL	TICL	CL_LD_	BS-CL_LD_	BL-CL_LD_	φ,β-Limit Value (%)
IR-64	16.70 a	14.55 a	48.89 a	11.22 a	4.48 a	11.82 ab	6.98 a	8.49 bcd	38.17 a	58.18 a
IR-42	17.22 a	14.74 a	49.92 a	11.36 a	4.86 a	12.60 b	7.36 a	8.78 cd	38.83 a	57.24 a
Brown	17.87 ab	15.23 b	50.05 a	12.08 b	4.48 a	11.53 ab	7.29 a	8.23 ab	37.67 a	59.20 b
Red	17.93 b	15.29 b	50.02 a	12.32 b	4.61 a	10.88 a	7.11 a	7.93 a	37.02 a	60.35 c
Black	18.32 b	15.54 b	50.52 a	12.38 b	4.94 a	12.60 b	7.44 a	8.76 d	36.78 a	59.40 b

Values of CL—BL-CL_LD_ are expressed as mean percentage (*n* = 2). Different letters on each column are significantly different (*p* < 0.05) from each other. According to one-way ANOVA. CL = average chain length of amylopectin; SCL = CL of short chains; LCL = CL of long chains; ECL = external chain length (CL × (φ,β-limit value/100) + 1.5); ICL = internal chain length (CL—ECL—1); TICL = total internal chain length (B-CL_LD—_1); CL_LD_ = average chain length of φ, β-limit dextrin; BS-CL_LD_ = CL_LD_ of short B-chains; BL-CL_LD_ = CL_LD_ of long B-chains; φ,β-limit value: calculated from the difference in CL of amylopectin and its φ,β-limit dextrin.

**Table 3:** Average chain lengths of A and B chains obtained by high-performance anion-exchange chromatography.

Sample	A-Chains ^1^	A_fp_^2^	A_crystal_^3^	B-Chains ^4^	BS	BL	B_fp_^5^	BS_major_^6^	B2 ^7^	B3 ^8^
IR-64	53.98 b	9.62 d	44.36 a	46.02 a	39.29 a	6.72 a	20.45 a	19.16 a	4.96 a	0.76 a
IR-42	53.84 b	8.65 c	45.19 a	46.16 a	38.72 a	7.44 a	19.06 a	20.00 a	4.91 a	0.82 a
Brown R	49.78 a	5.49 b	44.29 a	50.22 b	39.29 a	7.33 a	23.57 b	19.66 a	4.99 a	0.75 a
Red R	48.27 a	5.24 b	43.03 a	51.73 b	44.71 b	7.02 a	26.12 b	18.92 b	4.90 a	0.64 a
Black R	53.12 b	4.25 a	48.87 a	46.88 a	38.77 a	8.11 a	19.48 a	19.69 a	4.88 a	0.68 a

Values of A-chains—B3 are expressed as mean percentage (*n* = 2). Different letters on each column are significantly different (*p* < 0.05) from each other. According to one-way ANOVA. ^1^ detected as maltose after debranching φ,β-limit dextrins. ^2^ “Fingerprint” A-chains at DP 6−8 in the original amylopectin sample. ^3^ A-chain crystalline calculated as all the A-chains minus A_fp_. ^4^ B-chains correspond to DP > 3 in φ,β-limit dextrins and were divided into BS (short chains) and BL (long chains) with DP ≤ 22–24 and DP ≥ 23–25, respectively. ^5^ “Fingerprint” B-chains at DP 3–7 in φ,β-limit dextrins. ^6^ the major group of short B-chains at DP 22–24, depending on the sample. ^7^ long chains between DP 22 or 25 and 55, depending on the sample. ^8^ Long chains.

**Table 4:** Molar ratios of different chain categories of rice amylopectins and their φ,β-limit value.

Sample	A:B	S:L ^1^	BS:BL	A_crystal_:BS	A_crystal_:B	B_fp_:BS_major_
IR-64	1.17 a	14.92 b	5.85 ab	1.13 a	0.96 a	1.07 ab
IR-42	1.17 a	13.14 ab	5.20 ab	1.17 a	0.98 a	0.95 ab
Brown R	0.99 a	12.21 a	5.36 ab	1.13 a	0.88 a	1.20 a
Red R	0.93 a	12.16 a	6.37 b	0.96 a	0.83 a	1.38 b
Black R	1.13 a	11.59 a	4.78 a	1.26 a	1.04 a	0.99 a

Values of A:B—B_fp_:BS_major_ is expressed as mean percentage (*n* = 2). Different letters on each column are significantly different (*p* < 0.05) from each other. According to one-way ANOVA. ^1^ ratio of short and long chains in amylopectin.

## Data Availability

The original contributions presented in the study are included in the article, further inquiries can be directed to the corresponding author.
